# Evaluation of the effect of metformin and aspirin on utero placental circulation of pregnant women with PCOS

**Published:** 2012-05

**Authors:** Ashraf Jamal, Forozan Milani, Ashraf Al-Yasin

**Affiliations:** 1 *Department of Obstetrics and Gynecology, Tehran University of Medical Sciences, Tehran, Iran.*; 2 *Reproductive Health Research Center, Department of Obstetrics and Gynecology, Alzahra Hospital, Guilan University of Medical Sciences, Guilan, Iran.*

**Keywords:** *Metformin*, *Aspirin*, *PCOS*, *Uteroplacental circulation*

## Abstract

**Background:** Women with polycystic ovary syndrome (PCOS) often are infertile and even if they become pregnant, there are complications with some adverse outcomes. It has been reported that aspirin and metformin improve uteroplacental circulation and reduce pregnancy complications.

**Objective:** To determine and compare uteroplacental circulation and obstetrics complications in pregnant women with PCOS treated with metformin, aspirin and control group.

**Materials and Methods:** 105 pregnant women with PCOS were enrolled in this study after assessing uterine artery pulsatility index (PI) with Doppler ultrasonography at 12 weeks of gestation. The patients were divided into three groups and received metformin 2000 mg or aspirin 80 mg daily, or no intervention until the end of pregnancy. PI was assessed for the patients at 20 week of gestation and groups were followed up till delivery. PI and obstetrics complications such as gestational diabetes, preterm labor, preeclampsia and IUGR were compared among groups.

**Results:** All groups had significant reduction in the mean uterine artery PI at 20 weeks measurement (p<0.05), but this reduction was more in metformin and aspirin groups than control group (p=0.002). There was a significant difference in mean uterine artery PI 20 week of gestation in three groups (p=0.005). Adverse outcomes have seen 4 out of 35 in metformin group, 7 out of 35 in aspirin group and 11 out of 35 in control group. There weren’t significant differences among groups (p=0.12).

**Conclusion:** Metformin and low dose aspirin reduced uterine artery impedance but there was not associated with reduced obstetrics complication in women with PCOS.

## Introduction

Polycystic ovary syndrome (PCOS) is the most common cause of infertility in women in reproductive age. It occurs in 5-7% of reproductive age women. It is the most common form of female infertility in the United States. PCOS is a heterogeneous disorder of unclear etiology and important causes of both menstrual irregularity and androgen excess in women. It is characterized by oligomenorrhea, polycystic ovaries and hyperandrogenism (1-6). 

The PCOS women often are infertile and when they become pregnant, they are complicated with some adverse outcome of pregnancy such as first-trimester spontaneous abortion, preeclampsia, GDM (Gestational Diabetes Mellitus) and preterm labour (7-11). Preeclampsia and IUGR are to be consequences of impaired trophoblast invasion of maternal spiral arteries and increased impedance to flow and with result to abnormal uteroplacental blood flow (12). 

Doppler ultrasonography has become a valuable method for indirectly assessing utroplacental circulation from early gestation. When we study uterine artery, it can provide effective screening in identifying women of developing preeclampsia (13). When metformin is taken during the first trimester and throughout the pregnancy, it reduces obstetrics complication in pregnant women with PCOS and there is no evidence of an increased risk for major malformations (14). 

Beneficial effect of metformin doesn't seem to be related to reduce androgen levels, since a survey says that Metformin treatment in pregnant women with PCOS reduced pregnancy complication without influencing maternal androgen levels, but seems to reduce uterine artery impedance between 12-19 weeks of gestation (14, 15). In this manner, administered low dose aspirin in early pregnancy of women at risk of preeclampsia with abnormal uterine artery, Doppler may reduce risk of preeclampsia (16). 

And low dose aspirin appears to be useful in high risk women of developing preeclampsia and some clinicians recommend low dose aspirin for women at moderate or high risk for preeclampsia. No adverse maternal or fetal effects related to low dose aspirin have been reported (17-20). It has been reported that aspirin and metformin improved uteroplacental circulation and reduced pregnancy complications (15, 16). 

Therefore our randomized controlled clinical trial study was conducted to assess the effect of metformin on the uteroplacental circulation in comparison with aspirin and to determine pregnancy complications in pregnant women with poly cystic ovary syndrome.

## Materials and methods

This study was done with financial support of Tehran University of Medical Sciences. During the period from September 2008-2009, 105 pregnant women with PCOS were enrolled in the study. They were chosen from outpatient clinic of infertility at Shariati Hospital of Tehran University. 

After an ultrasound scanning confirming alive singleton embryo at 6-12 weeks of gestation patients were entered in the study. They gave written consent and were assessed for uterine artery PI with Doppler Ultrasonography at 12^th^ week of gestational age. The inclusion criteria were: (a) diagnosis of PCOS before the actual pregnancy; (b) age 18-40 years; (c) gestational age 6-12 weeks; and (d) a single viable Fetus and no history of diabetes mellitus or hypertension. 

PCOS diagnosis before pregnancy was based on the presence of poly cystic ovaries verified by ultrasonography (more than eight subcapsular follicles with a diameter of 3-9mm) and oligomenorea (length of menstrual cycle more than 35 days or hirsutism or testosterone >2 nmol/L and all women took metformin before entering the study. Patients were randomly assigned into three groups of 35 patients by a computer program and coincidence numbers arrangement are hidden in sealed envelopes that were calculated upon them. Each patient took aspirin (one tablets 80 mg daily) or Metformin, (two tables twice daily 2000mg) or no intervention till the end of pregnancy. 

All participants received one ferrous sulfate tablet, 1 mg folate and one multivitamin tablet daily for the rest of pregnancy. Pulsed wave Doppler ultrasound examinations of the uterine. Arteries supported by directional color Doppler were performed at 12 and 20 week of gestation. We used the color Doppler ultrasound machine (Acusan *Antares* Siemens) equipped with a 6.5-MHz transvaginal transducer and a 3.5-MHz transabdominal transducer. 

At the 12-week examination the uterine arteries were examined lateral to the cervix at the level of the internal os, using a transvaginal transducer. From the 20-week examination uterine arteries were examined using a transabdominal transducer. The uterine arteries were located in an oblique plane of the pelvis to identify the left and right uterine arteries at the level of internal os with color flow mapping then pulsed-wave Doppler was used to obtain three similar consecutive waveforms and PI was measured. 

Three measurements of each uterine artery were performed. The mean PI of each uterine artery and the bilateral mean PI for each gestational age was calculated. All ultrasound examinations were performed by single person of the team. The study was a single blind, controlled trial and the examiner was blinded to group status during the ultrasound examinations. The results of Doppler examination of the uterine arteries were not available to the clinicians. 

All pregnant women were observed and treated during pregnancy according to standard antenatal care and were followed to the end of pregnancy. In addition, (Fasting Blood Glucose) FBS was measured on all women at first visit and Glucose Challenge Test (GCT) with 50 gr Glucose was measured at 24 and 28 gestational weeks. If GCT was more than 130 then Oral Glucose Tolerance Test (OGTT) with 100gr Glucose was performed. Insulin therapy was recommended when standard dietary management was not consistently insufficient for GDM treatment. Pre-eclampsia was defined as a blood pressure ≥140/90 mmHg with concomitant albuminuria ≥0*.*3 g/24 h. 

Preterm delivery was defined as delivery before 37 gestational weeks based on first-trimester ultrasound scan. IUGR refers to a weight below the 10th percentile for gestational age. Adverse outcome included preterm labor, GDM, IUGR and preeclampsia.

**Statistical analysis**

Data were analyzed using SPSS version16. The normality of continuous data was checked by One Sample Kolmogorov-Smirnov Test, and between G groups comparison was done by a one way ANOVA and Post Hoc Tukey HSD test. The categorical outcome variables were compared with chi-square or Fisher’s exact test. Paired t-test was used to compare the change of mean in uterine artery PI measured at 12 and 20 weeks of gestation within groups. The statistical significant was set at 0.05 level.

## Results

**Figure 1:**
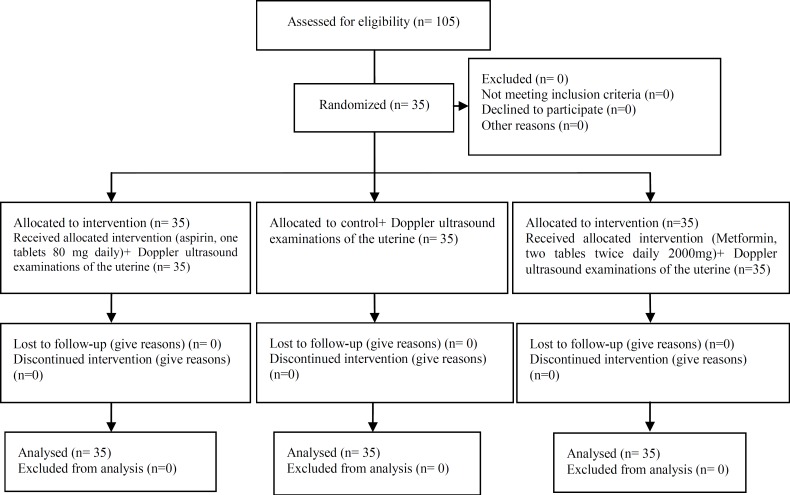
Consort flow chart of RCT

**Figure 2:**
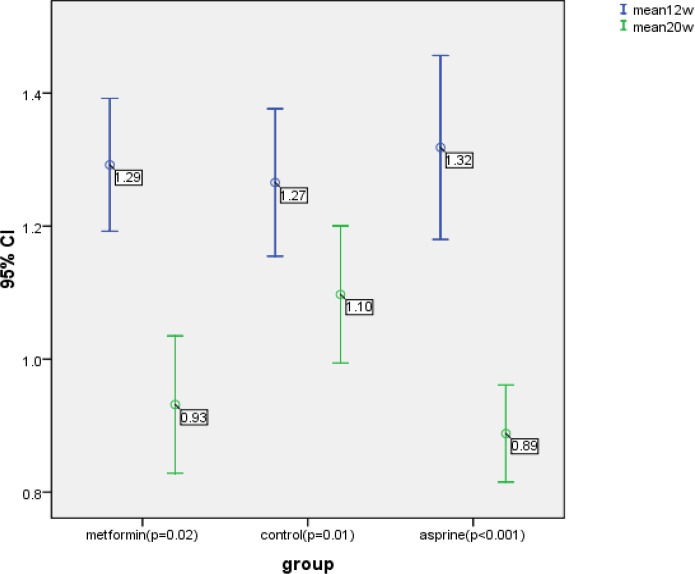
The mean uterine artery PI measured at 12 and 20 weeks of gestation in three study groups

One hundred five patients were eligible and divided into three groups. Three groups were homogenous according to the mean of age, BMI, gravidity (Figure 1). They had non-significant mean bilateral uterine artery PI at 12 weeks of gestation, (p=0.813) (Table I). All three groups had significant reduction in the mean uterine artery PI at 20 weeks measurement (p<0.05) (Figure 2), but this reduction was more pronounced in metformin and aspirin groups than the controls (0.36 and 0.43 versus 0.17, respectively, p=0.002) (Table I). There was a statistically significant difference in mean uterine artery PI at 20 weeks of gestation in three groups (p=0.005); and aspirin and metformin group had significantly lower mean uterine artery PI compared to the control group (p=0.005 and p=0.035, respectively) but there was no significant difference in mean PI between asprin and metformin (p=0.785).

Adverse outcome including preeclampsia, Diabetes, IUGR and preterm labor occurred 4 of 35 in metformin group 7 of 35 in asprine group and 11 of 35 in the control group. The aspirin and control groups had more chance for developing any adverse outcome comparing to metformin group, but there were not significant relation between these groups (p=0.12). Eight patients, 2 (5.7%) of metformin, 2 (5.7%) of Aspirin and 4 (11.4%) of control groups, developed preeclampsia, although the chance of preeclampsia was 2 times more in control group comparing to metformin or aspirin groups, but it was not statistically significant. (p=0.58). Metformin group had the lowest (5.7%) and control group had the highest (14.3%) rate of preterm deliver. The difference was not statistically significant (p=0.47). 

Three women developed GDM in metformin group who were controlled by diet. In the control and aspirin groups respectively 6 and 5 women developed GDM that 3 of them in each group controlled by diet and the others controlled by insulin. The chance of GDM in metformin was lower than other groups, although it was not statistically significant (p=0.56). Two fetuses in control and one in aspirin group were IUGR (p=0.36). The infant birth weight was 3265+366 gr in metformin, 3329+537 gr in aspirin and 3104+359 gr in control groups, but it was not statistically significant (p=0.08) (Table II).

**Table I:** Patients' sonographic characteristics in study groups

	**Metformin group (n=35)**	**Aspirin group (n=35)**	**Control group (n=35)**	**p-value**
MUAPI at 12 weeks of gestation	1.29 (+0.29)	1.32 (+0.40)	1.27 (+032)	0.813
MUAPI at 20 weeks of gestation	0.93 (+0.30)#	0.89 (+0.21)	1.10 (+0.30)*	0.005
MUAPI 20- MUAPI 12	-0.36 (+0.33)	-0.43 (+0.29)	-0.17 (+0.33)	0.002

MUAPI: Mean uterine artery pulsatility index.

* p= 0.005 compare to aspirin group and p= 0.035 compare to metformin group.

# p= 0.785 compare to aspirin group.

**Table II:** Comparison of pregnancy outcomes between study groups

	**Metformin group (n=35)**	**Aspirin group (n=35)**	**Control group (n=35)**	**p-value**
Preeclampsia	2 (5.7)	2 (5.7)	4 (11.4)	0.58
Preterm delivery	2 (5.7)	3 (8.6)	5 (11.4)	0.47
Gestational diabetes	3 (8.6)	5 (14.3)	6 (17.1)	0.56
IUGR	0	1 (2.9)	2 (5.7)	0.36
Any adverse outcome	4 (11.4)	7 (21)	11 (31.4)	0.12
Infant birth weight	3265+366	3329+537	3104+359	0.08

## Discussion

All three groups had significant reduction in the mean uterine artery PI at 20 weeks of gestation (p<0.05), but this reduction was more in metformin and aspirin groups than control group, (p=0.002). Salvesen and his colleague’s study showed that women treated with metformin had a statically significant reduction in mean bilateral uterine artery PI between 12 and 19 weeks compared with women in placebo group (0.03). Our findings indicate that metformin and aspirin reduces uterine artery impedance and they may influence the uteroplacental circulation. Therefore the improved uterine artery flow between 12 and 20 weeks conform to a hypothesis of a positive effect of metformin and aspirin in trophoblast invasion in women with PCOS.

In some studies, pregnancy complications such as diabetes controlled with insulin, abortion and IUGR in pregnant women of PCOS treated with metformin had a significant reduction in comparison with control group (21-23). In Vankye’s study metformin treatment from first trimester to delivery in women with PCOS compared to placebo group didn’t reduced pregnancy complications (28) but in Salvesen’s study severe pregnancy complications in pregnant women with PCOS treated with metformin was significantly reduced compared to the placebo group (15). 

Our study shows that severe pregnancy complications in women with PCOS treated with metformin was fewer than aspirin and control group and they weren’t significant differences among groups (p=0.12). Gestational diabetes is related to resistance and increasing insulin during pregnancy. A considerable percent of women with PCOS display insulin resistance and elevated Insulin level. Some studies have demonstrated since metfomin had a reassuring safety during pregnancy that metformin reduces hyperinsulinism, Improve ovulation and decrease serum testosterone concentration in women with PCOS (24-27) and Glueck’s study and coworkers conclude that the use of metformin in PCOS women was associated with 10 fold reduction in gestational diabetes (31% to 3%) and it reduces insulin resistance (28). My survey showed that diabetes in metformin group was lower than aspirin and control groups. These findings support the hypothesis that insulin sensitivity seem to have improved in the metformin group and metformin induces the reduction insulin blood level in pregnant women with PCOS.

In some studies experience a higher rate of preeclampsia in women with PCOS (29-31). And Allaa Ebrashy and Bujld’s studies show that Low dose aspirin administered in early weeks of pregnancy to pregnant women with high risk of preeclampsia and abnormal uterine Doppler findings, may reduce severe preeclampsia (16, 32). Bujold and coworker, in randomized controlled trials of pregnant women at risk of preeclampsia who were assigned to receive aspirin or placebo concluded that low dose aspirin initiated in early pregnancy is an efficient method of reducing the incidence of preeclampsia and IUGR (33).

In our study preeclampsia in control group was 2-fold metformin and aspirin groups but the rate of preeclampsia was not difference between metformin and aspirin groups. Uterine artery flow impedance in pregnant women treated with Aspirin and metformin in comparison with control group had significantly reduction and also this reduction was better in aspirin group than other groups. Pregnancy complication such as IUGR and preeclampsia are thought to be consequences of impaired trophoblastic invasion of maternal arteries.

Our study has demonstrated that aspirin improved uteroplacental circulation better than other groups. Therefore it can be postulated that if pregnant women with PCOS with abnormal uterine artery PI in first trimester treated with low dose aspirin, rate of pregnancy complication such as preeclampsia and IUGR would become the lower than other groups. Metformin and aspirin can both significantly improve uteroplacental blood flow and they have fewer adverse outcomes in comparison with control group but the difference was not statistically significant. Since metformin and Aspirin are safe and inexpensive, larger clinical studies are required to ensure the efficacy of these drugs in pregnancy. Therefore, In order to prove the effect of treatment with metformin or Aspirin in prevention of complication in pregnant women with PCOS, more randomized clinical trial studies would be needed. 

## Footnote

***Registration ID in IRCT: IRCT138812123485N1***
